# Evolution and mitigation strategies of online public opinion: An analysis using an improved replicator dynamic three-party game model

**DOI:** 10.1371/journal.pone.0325744

**Published:** 2025-07-08

**Authors:** Jiadong Chen, Jie Xin, Wan Ni

**Affiliations:** 1 School of Journalism and Communication, Shandong University, Jinan, China; 2 School of Archaeology, Shandong University, Jinan, China; Sustainable Psychological Science, AUSTRALIA

## Abstract

In the digital age, online public opinion plays a pivotal role in shaping social stability, policymaking, and public trust in institutions. Given the frequent occurrence of public opinion crises, it is imperative to explore their evolutionary dynamics and effective mitigation strategies. This study develops a three-party evolutionary game model involving the government, ordinary netizens, and media/KOLs, incorporating both inter-group strategy influence and intra-group incentive effects. The model enhances traditional replicator dynamics by embedding incentive coefficients that reflect the strategic suppressive or promotive effects within each group. Simulation results reveal that changes in incentive structures significantly affect the speed and stability of opinion convergence. For instance, when the media’s suppression of dissemination strategies is strong ($\lambda_{3}=1.5$), all groups reach near-equilibrium within 3–5 time steps, with netizen participation stabilizing above 0.99 by *t* = 3. However, when only the government’s suppressive influence increases ($\lambda_{1}$), convergence is slower and displays diminishing returns. As $\lambda_{1}$ continues to rise, netizen responsiveness plateaus, indicating a saturation effect whereby excessive suppression loses effectiveness in accelerating stabilization. These findings challenge the assumption that earlier or stronger intervention is inherently more effective. Instead, they underscore the importance of calibrated timing and intensity, as public sentiment evolves through the interplay of government response, media coordination, and audience receptiveness. Netizens respond more rapidly than institutional actors, reflecting their sensitivity to perceived information gaps. Effective mitigation of negative sentiment thus requires not only timely action but also adaptive adjustment of strategic influence in accordance with systemic feedback.

## 1 Introduction

Understanding the evolution of online public opinion has become a prominent interdisciplinary issue in communication studies, computational social science, and public governance research [1,2]. As digital platforms reshape how individuals access, interpret, and respond to information [3,4], the mechanisms underlying collective opinion formation are increasingly viewed as dynamic, multi-agent processes [5]. Existing models—ranging from agenda-setting theories to diffusion frameworks—have provided valuable insights into specific actors or channels [6–8]. However, few studies have systematically integrated the strategic interplay among governments [9], media/KOLs [10], and ordinary netizens [11] within a unified analytical framework. This gap limits our ability to predict, simulate, or guide opinion dynamics in complex and rapidly changing environments.

In particular, while prior studies have examined government intervention timing [12], media influence [6], and netizen behavior [13], these factors are frequently analyzed in isolation [11]. Moreover, much of the existing modeling work assumes fully rational agents operating with complete information. In practice, however, public opinion actors make decisions under uncertainty, time constraints, and limited cognitive resources. This condition—known as bounded rationality [14]—better reflects real-world decision-making environments, where individuals rely on heuristic shortcuts and context-driven judgments [15–17].

To better capture these complexities, this study develops a comprehensive evolutionary game model that incorporates the strategies of governments, media/KOLs, and netizens. By improving traditional replicator dynamics with the introduction of incentive coefficients, the model reflects bounded rationality and intra-group strategic dependencies, enabling the simulation of various combinations of strategic choices and their effects on the stability of public opinion. Beyond its theoretical contribution, the model offers practical value for public opinion governance by supporting evidence-based analysis of intervention timing, media engagement, and participatory dynamics. It provides a useful analytical tool for policymakers seeking to respond effectively to evolving online sentiments while preserving institutional credibility and fostering constructive discourse.

The remainder of this paper is organized as follows: Sect 2 reviews the relevant literature and theoretical foundations. Sect 3 presents the construction of the three-party game model and analyzes the evolutionary stable strategies (ESS) of each actor. Sect 4 introduces an improved replicator dynamic equation and compares it with existing models. Finally, Sect 5 discusses the findings and their implications for online public opinion governance.

## 2 Literature review and theoretical basis

### 2.1 Actors in the online public opinion ecosystem

Public opinion in the digital age is shaped by complex interactions among governments, media/KOLs, and netizens [18]. Traditional communication theories, such as agenda-setting [19] and framing [20], emphasize the role of media in shaping public discourse by selectively highlighting and contextualizing information. However, with the advent of digital platforms, this top-down structure has evolved into a decentralized, engagement-driven ecosystem [21,22]. Platforms now amplify content based on algorithmic engagement metrics rather than editorial gatekeeping [23], enabling KOLs and ordinary users to significantly shape public narratives [24]. These transformations have fostered phenomena such as echo chambers [25], viral misinformation [26], and rapid emotional shifts [27], which contribute to the fluidity and unpredictability of online discourse [28].

Government agencies, in response to evolving public opinion, have adopted diverse strategies including crisis communication [29], information regulation [30], and platform engagement [31]. Timely and transparent responses are found to mitigate disinformation or misinformation [32], while delayed or non-transparent responses can lead to public backlash [33]. Recent studies also suggest that strategic collaboration with trusted media and KOLs can improve message credibility [34,35], though excessive intervention risks undermining trust [36,37].

Media and KOLs act as both disseminators and amplifiers of discourse. Their influence is shaped by ideological [38], commercial [39], or personal [40] motives, introducing unpredictability into information framing [41,42]. The two-step flow model [43] remains relevant for understanding how information spreads from institutions to the public via opinion leaders. Netizens, once considered passive receivers, now actively participate in agenda-setting and emotional mobilization. Social identity theory [44] explains how individuals align with narratives based on group affiliation, leading to polarization and collective action, while emotional contagion studies show that high-arousal content spreads faster and intensifies sentiment clustering [45]. These developments underscore the need for a holistic framework that integrates the dynamic strategies of all three actors. Online discourse is not a linear sequence of messages but a networked, adaptive system in which actors continuously respond to one another’s behavior [18].

### 2.2 Theoretical foundations: Strategic behavior and bounded rationality

A key limitation of existing public opinion models is their reliance on static assumptions and rational-actor theory. In contrast, real-world discourse evolves under conditions of uncertainty, limited information, and fluctuating incentives. Strategic behavior among stakeholders is shaped not only by preferences and beliefs, but also by perceptions of others’ responses. This interdependent decision-making process calls for analytical tools that can model adaptation over time.

Bounded rationality provides a foundational lens through which to understand these decisions. Unlike classical models that assume perfect optimization, bounded rationality acknowledges cognitive constraints and reliance on heuristics [14–17]. These limitations are especially pronounced in fast-moving online environments, where actors must react quickly to shifting narratives and emotional stimuli. For governments, media, and netizens alike, decision-making is often satisficing rather than optimizing—a dynamic shaped by both internal constraints and the evolving behavior of others.

### 2.3 Modeling public opinion dynamics: From isolated agents to strategic interdependence

Evolutionary Game Theory (EGT) [46] provides a robust framework for capturing the adaptive and strategic nature of public opinion formation. It enables researchers to model how actors iteratively update their strategies based on payoffs and social feedback in dynamic environments. Recent applications have demonstrated its value in analyzing government intervention strategies [47], media narrative evolution [48], and netizen participation behaviors [49]. These studies collectively highlight the importance of modeling stakeholder adaptation in response to evolving incentive structures and contextual uncertainties [50].

In parallel, recent agent-based models (ABMs) have provided valuable micro-level insights into behavioral heterogeneity and localized interaction dynamics under conditions of public stress, such as panic buying during urban crises [51] and household energy-efficiency retrofit decisions in social networks [52]. While ABMs excel at simulating individual-level behaviors and emergent outcomes within complex networks, there remains a complementary need for meso-level theoretical models that capture the strategic interdependence among institutional actors and the systemic effects of incentive regulation in public opinion governance.

Despite these advances, existing EGT-based models often treat actors as isolated decision-makers, neglecting the strategic interdependence and feedback structures that characterize real-world public opinion dynamics. The lack of integrated frameworks to model multi-actor interactions and institutional responses has been identified as a key limitation [11]. To address this gap, the present study introduces a three-party evolutionary game model based on an improved replicator dynamic equation with embedded incentive coefficients. This enhancement enables the simulation of both inter-group strategic adjustments and intra-group suppressive dynamics among governments, netizens, and media/KOLs. By modeling how actors adapt their strategies in response to shifting costs, benefits, and regulatory delays, the framework offers a more realistic and interpretable representation of online opinion evolution. MATLAB-based simulations further demonstrate how variations in incentive structures influence the convergence trajectories of public sentiment, providing actionable insights for optimizing discourse governance in complex digital environments.

## 3 The evolutionary model of the three-party game

### 3.1 Establishing the payoff matrix for the three parties

In the game, the decision-making agents serve as the primary players. Ordinary netizens act as initiators, participants, and contributors in online public opinion events [53]. The outbreak of public discourse is often closely tied to government actions—particularly in moments when declining governmental credibility catalyzes public unrest [54]. Additionally, the trajectory of online opinion is influenced by media outlets and key opinion leaders (KOLs), who, through agenda-setting or the dissemination of biased or false information, can significantly shape public discourse [55].

While media and KOLs are treated as distinct actors in the model, their real-world behavior often evolves in response to content initially generated by netizens. KOLs commonly amplify or reframe such content, adding commentary that can steer sentiment in particular directions. For analytical tractability, however, this study models netizens and media/KOLs as behaviorally independent agents. This abstraction facilitates a clearer examination of intra-group dynamics, though it inevitably omits certain feedback processes present in actual communication ecosystems. Therefore, this study identifies three primary agents in the evolutionary game of online public opinion: the government, ordinary netizens, and media/KOLs.

A strategy refers to the options selected by each agent during the game. In the evolutionary game of online public opinion, the strategy sets for the three parties are as follows:

- The government’s strategy set: guide, not guide,
- The ordinary netizens’ strategy set: participate, not participate,
- The media and KOLs’ strategy set: spread, not spread.

**Fig 1:**
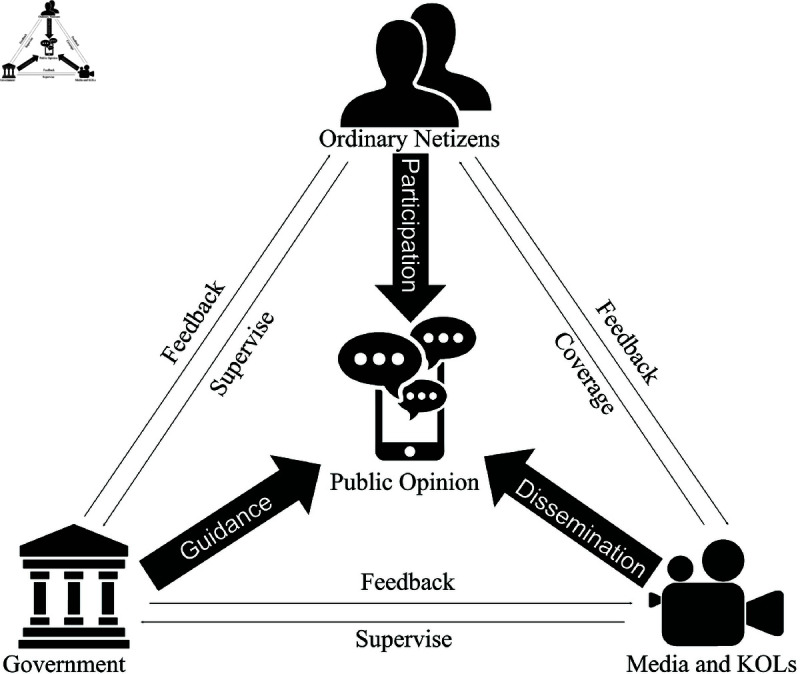
Relationship diagram of government, ordinary netizens, and media/KOLs.

**Fig 2:**
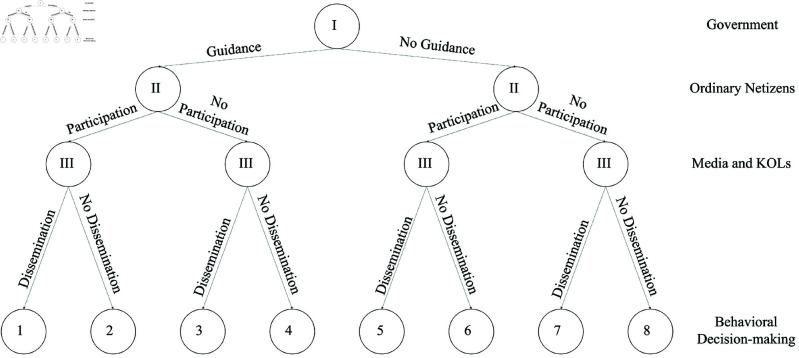
“Government-ordinary netizens-media and KOLs” three-party game tree model.

The diagram illustrating the three-party game relationship is shown in Fig 1, and the three-party game tree model for “government-ordinary netizens-media and KOLs” is presented in Fig 2.

The payoff in the game process refers to the expected outcomes for each agent resulting from their decisions at the conclusion of the game. Based on the parameters proposed by Liang Yanru and Liu Yiqing (2019), as well as the actual dynamics of online public opinion, the parameters for each agent are defined in terms of *Cost*, *Lose*, and *Profit*. The corresponding parameter values for the agents are shown in Table 1.

**Table 1:** Definition of parameters for each agent in public opinion events.

Agent	Parameter	Definition and examples	Notes
**Government** Let the delay parameter *a* represent the extent to which the government’s initial information release lags behind the other two agents when guidance is implemented.	*C*_*11*_	The cost of government guidance includes the time, manpower, effort, and financial resources, etc.	
	*C*_*12*_	The cost of government not providing guidance includes the loss of credibility caused by information asymmetry.	
	*L*_*11*_	The losses incurred by the government when it does not provide guidance include damage to the government’s image, loss of credibility, and increased pressure to manage public opinion.	
	*L*_*12*_	The losses caused by media and KOLs when the government does not provide guidance include the spread of negative public sentiment among the populace, as well as damage to the government’s image and credibility.	
	*L*_*13*_	The losses incurred by the government when providing guidance include public skepticism and antagonistic sentiment, as well as the intensification of opposition in public opinion.	
	*L*_*14*_	The losses caused by ordinary netizens when the government does not provide guidance, *L*_*14*_ ∈ *L*_*11,12*_.	Noted as 0.
	*P*_*11*_	The benefits gained by the government when providing guidance include enhanced governmental influence, improved credibility, and increased public trust.	
	*P*_*12*_	The benefits brought by media and KOLs when the government provides guidance include increased influence and broader dissemination of government information.	
	*P*_*13*_	The benefits brought by ordinary netizens when the government provides guidance, *P*_*13*_ ∈ *P*_*11,12*_.	Noted as 0.
	*P*_*14*_	The benefits the government gains by not providing guidance.	Noted as 0.
**Ordinary Netizens**	*C*_*21*_	The cost of participation for ordinary netizens includes the time, equipment, and effort, etc.	
	*C*_*22*_	The cost of non-participation for ordinary netizens.	Noted as 0.
	*L*_*21*_	When the government does not provide guidance, the losses incurred by ordinary netizens participating include exposure to false information and the spread of negative emotions.	
	*L*_*22*_	When the government provides guidance, the losses incurred by ordinary netizens participating include the risk of online harassment due to comments made based on misunderstandings of the event.	
	*L*_*23*_	The losses incurred by ordinary netizens when they do not participate (regardless of whether the government provides guidance).	Noted as 0.
	*P*_*21*_	The benefits gained by ordinary netizens when they participate (regardless of whether the government provides guidance) include access to relevant information and opinions that can serve as topics for discussion.	
	*P*_*22*_	The benefits gained by ordinary netizens from media and KOLs when they participate (regardless of whether the government provides guidance) include perspectives provided by media and KOLs, as well as a synthesized understanding of the event’s causes and consequences curated by these sources.	
	*P*_*23*_	The benefits gained by ordinary netizens from government guidance when they participate include official event updates and guidance provided by relevant authorities.	
	*P*_*24*_	When the government does not provide guidance, the benefits gained by ordinary netizens through participation.	Noted as 0.
**Media and KOLs**	*C*_*31*_	The costs for media and KOLs in spreading information include the time, effort, and resources spent on marketing and promoting the content.	
	*C*_*32*_	The costs for media and KOLs in not spreading information.	Noted as 0.
	*L*_*31*_	The losses for media and KOLs when not spreading information (regardless of whether the government provides guidance) include reduced traffic and decreased opportunities for monetizing data.	
	*L*_*32*_	When the government provides guidance, the losses for media and KOLs in spreading information include oversight from cybersecurity authorities, platform warnings or interviews, and potential backlash from ordinary netizens, which may lead to loss of followers.	
	*L*_*33*_	When the government does not provide guidance, the losses for media and KOLs in spreading information, *L*_*33*_ ∈ *L*_*32*_.	Noted as 0.
	*P*_*31*_	The benefits for media and KOLs in spreading information (regardless of whether the government provides guidance) include increased traffic and opportunities for data monetization.	
	*P*_*32*_	When the government provides guidance, the benefits for media and KOLs in spreading information include reduced regulatory pressure and a lower risk of online harassment from ordinary netizens.	
	*P*_*33*_	When the government does not provide guidance, the benefits for media and KOLs in spreading information, *P*_*33*_ ∈ *P*_*31*_.	Noted as 0.

Based on the parameters in Table 1, the payoff matrix for each agent under different strategic behaviors can be calculated. The resulting payoff matrix is presented in Table 2.

**Table 2:** Payoff matrix for the three-party game in public opinion events.

Strategies of game participants		Government guidance (*x*)	Government does not guide (1-*x*)
Ordinary Netizens Participate (*y*)	Media and KOL Spread Information (*z*)	(1-a)(P_11_+P_12_-C_11_-L_13_) P_21_+P_22_+P_23_-C_21_-L_22_ P_31_+P_32_-C_31_-L_32_	-C_12_-L_11_-L_12_ P_21_+P_22_-C_21_-L_21_ P_31_-C_31_
	Media and KOL Do Not Spread Information (1-*z*)	(1-a)(P_11_-C_11_-L_13_) P_21_+P_23_-C_21_-L_22_ -L_31_	-C_12_-L_11_ P_21_-C_21_-L_21_ -L_31_
Ordinary Netizens Do Not Participate (1-*y*)	Media and KOL Spread Information (*z*)	(1-a)(P_12_-C_11_-L_13_) 0 P_31_+P_32_-C_31_-L_32_	-C_12_-L_12_ 0 P_31_-C_31_
	Media and KOL Do Not Spread Information (1-*z*)	-C_11_-L_13_ 0 0	-C_12_ 0 0

### 3.2 Solving the evolutionary stable strategy

#### 3.2.1 Construction of the payoff expectation function.

**(1) Government’s Payoff Expectation Function**

Expected Payoff for the Government When Choosing the “Guidance” Strategy:

$$U_{11}=(1-a)(yP_{11}+zP_{12})+[a(y+z-yz)-1](C_{11}+L_{1}3)$$ (1)

Expected Payoff for the Government When Choosing the “No Guidance” Strategy:

$$U_{12}=-yL_{11}-zL_{12}-C_{12}$$ (2)

Average Expected Payoff for the Government:

$${\bar{U}}_{1}=(1-a)(yP_{11}+zP_{12})+[a(y+z-yz)-1](C_{11}+L_{13})$$ (3)

**(2) Payoff Expectation Function for Ordinary Netizens**

Expected Payoff for Ordinary Netizens When Choosing the “Participate” Strategy:

$$U_{21}=x(P_{23}+L_{21}-L_{22})+zP_{22}+P_{21}-C_{21}-L_{21}$$ (4)

Expected Payoff for Ordinary Netizens When Choosing the “Not Participate” Strategy:

$$U_{22}=0$$ (5)

Average Expected Payoff for Ordinary Netizens:

$${\bar{U}}_{2}=y[x(P_{23}+L_{21}-L_{22})+zP_{22}+P_{21}-C_{21}-L_{21}]$$ (6)

**(3) Payoff Expectation Function for Media and KOLs**

Expected Payoff for Media and KOLs When Choosing the “Spread” Strategy:

$$U_{31}=P_{31}-C_{31}+x(P_{32}-L_{32})$$ (7)

Expected Payoff for Media and KOLs When Choosing the “Not Spread” Strategy:

$$U_{32}=-yL_{31}$$ (8)

Average Expected Payoff for Media and KOLs:

$${\bar{U}}_{3}=z(1-z)[P_{31}-C_{31}+x(P_{32}-L_{32})+yL_{31}]$$ (9)

#### 3.2.2 Solving the evolutionary stable strategy using replicator dynamics.

Replicator Dynamic Equation for the Government:

$$F(x)=\frac{dx}{dt}\;=x\cdot \left(U_{11}-{\bar{U}}_{1}\right)\;=x(1-x)\{z[a\cdot (C_{11}+L_{13})(1-y)+(1-a)P_{12}+L_{12}]\;\;+y[(1-a)P_{11}+L_{11}]+(ay-1)(C_{11}+L_{13})+C_{12}\}.$$ (10)

Replicator Dynamic Equation for Ordinary Netizens:

$$F(y)=\frac{dy}{dt}\;=y\cdot \left(U_{21}-{\bar{U}}_{2}\right)\;=y(1-y)[zP_{22}+x\cdot \left(P_{23}+L_{21}-L_{22}\right)+P_{21}-C_{21}-L_{21}].$$ (11)

Replicator Dynamic Equation for Media and KOLs:

$$F(z)=\frac{dz}{dt}\;=z\cdot \left(U_{31}-{\bar{U}}_{3}\right)\;=z(1-z)[P_{31}-C_{31}+x\cdot \left(P_{32}-L_{32}\right)+y\cdot L_{31}].$$ (12)

By combining (Eq 10) to (12), the replicator dynamic system for the government, ordinary netizens, and media/KOLs can be obtained, denoted as Eq (13).

**Analysis of the Government’s Stable Strategy**

When


$$z=\frac{y[(1-a)P_{11}+L_{11}]+(ay-1)(C_{11}+L_{13})+C_{12}}{a(C_{11}+L_{13})(y-1)-(1-a)P_{12}-L_{12}},$$

then


$$F(x)=\frac{dx}{dt}\equiv 0,$$

and ∀x∈[0,1], both strategies (“guidance” and “no guidance”) are stable strategies for the government.

When


$$z\neq \frac{y[(1-a)P_{11}+L_{11}]+(ay-1)(C_{11}+L_{13})+C_{12}}{a(C_{11}+L_{13})(y-1)-(1-a)P_{12}-L_{12}},$$

let $F(x)=\frac{dx}{dt}=0$, and solve for *x*. The solutions are:


x=0andx=1,

representing two stable states for *x*.

When


$$z>\frac{y[(1-a)P_{11}+L_{11}]+(ay-1)(C_{11}+L_{13})+C_{12}}{a(C_{11}+L_{13})(y-1)-(1-a)P_{12}-L_{12}},$$

$${\frac{dF(x)}{dx}|}_{x=0}>0\;\text{and}\;{\frac{dF(x)}{dx}|}_{x=1}<0.$$

Therefore, *x* = 1 is the equilibrium point, and the government choosing the “guidance” strategy becomes the evolutionary stable strategy.

When


$$z<\frac{y[(1-a)P_{11}+L_{11}]+(ay-1)(C_{11}+L_{13})+C_{12}}{a(C_{11}+L_{13})(y-1)-(1-a)P_{12}-L_{12}},$$

$${\frac{dF(x)}{dx}|}_{x=0}<0\;\text{and}\;{\frac{dF(x)}{dx}|}_{x=1}>0.$$

Therefore, *x* = 0 is the equilibrium point, and the government choosing the “no guidance” strategy becomes the evolutionary stable strategy.

**Fig 3:**
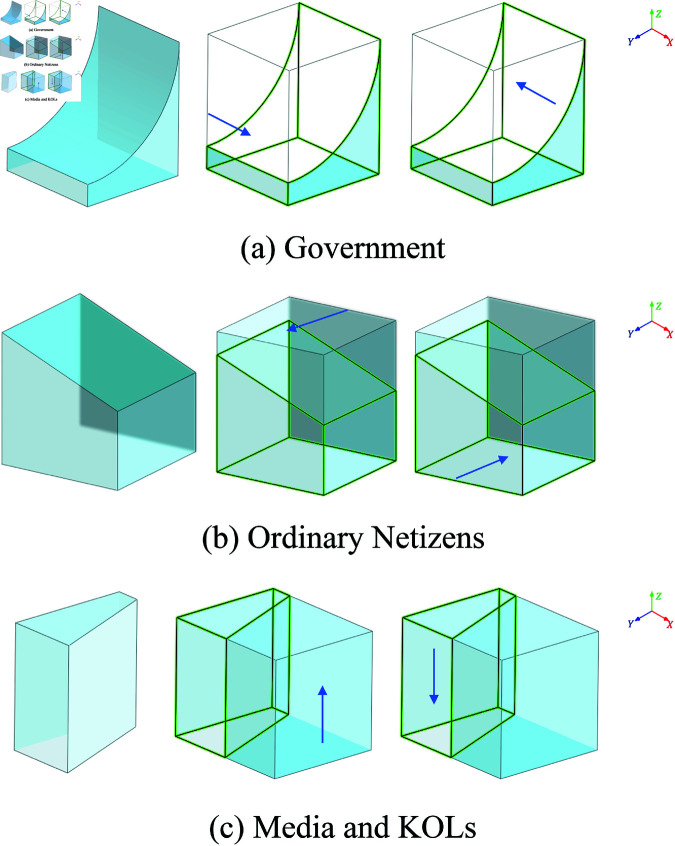
Diagram of the evolutionary stable strategy (ESS) dynamics. Each subfigure depicts the directional dynamics of the system under three initial conditions—where the corresponding strategy probability is equal to (left), greater than (middle), or less than (right) its ESS value. The 3D surfaces represent the state space, with arrows indicating the direction of strategic adjustment. Arrows aligned with the positive axis reflect reinforcement of the dominant strategy, while those in the opposite direction indicate reversal toward the competing strategy.

The dynamic changes in the government’s strategy under different conditions are illustrated in Fig 3a.

**Analysis of the Stable Strategy for Ordinary Netizens**

When


$$z=\frac{C_{21}+(1-x)L_{21}-xL_{22}-xP_{23}-P_{21}}{P_{22}},$$

then


$$F(y)=\frac{dy}{dt}\equiv 0,$$

and ∀y∈[0,1], both strategies (“participate” and “not participate”) are stable strategies for ordinary netizens.

When


$$z\neq \frac{C_{21}+(1-x)L_{21}-xL_{22}-xP_{23}-P_{21}}{P_{22}},$$

let $F(y)=\frac{dy}{dt}=0$, and solve for *y*. The solutions are:


y=0andy=1,

representing two stable states for *y*.

When


$$z>\frac{C_{21}+(1-x)L_{21}-xL_{22}-xP_{23}-P_{21}}{P_{22}},$$

$${\frac{dF(y)}{dy}|}_{y=0}>0\;\text{and}\;{\frac{dF(y)}{dy}|}_{y=1}<0.$$

Therefore, *y* = 1 is the equilibrium point, and ordinary netizens choosing the “participate” strategy becomes the evolutionary stable strategy.

When


$$z<\frac{C_{21}+(1-x)L_{21}-xL_{22}-xP_{23}-P_{21}}{P_{22}},$$

$${\frac{dF(y)}{dy}|}_{y=0}<0\;\text{and}\;{\frac{dF(y)}{dy}|}_{y=1}>0.$$

Therefore, *y* = 0 is the equilibrium point, and ordinary netizens choosing the “not participate” strategy becomes the evolutionary stable strategy.

The dynamic changes in the strategies of ordinary netizens under different conditions are illustrated in Fig 3b.

**Analysis of the Stable Strategy for Media and KOLs**

When


$$x=\frac{C_{31}-P_{31}-yL_{31}}{P_{32}-L_{32}},$$

then


$$F(z)=\frac{dz}{dt}\equiv 0,$$

and ∀z∈[0,1], both strategies (“spread” and “not spread”) are stable strategies for media and KOLs.

When


$$x\neq \frac{C_{31}-P_{31}-yL_{31}}{P_{32}-L_{32}},$$

let $F(z)=\frac{dz}{dt}=0$, and solve for *z*. The solutions are:


z=0andz=1,

representing two stable states for *z*.

When


$$x>\frac{C_{31}-P_{31}-yL_{31}}{P_{32}-L_{32}},$$

$${\frac{dF(z)}{dz}|}_{z=0}>0\;\text{and}\;{\frac{dF(z)}{dz}|}_{z=1}<0.$$

Therefore, *z* = 1 is the equilibrium point, and media and KOLs choosing the “spread” strategy becomes the evolutionary stable strategy.

When


$$x<\frac{C_{31}-P_{31}-yL_{31}}{P_{32}-L_{32}},$$

$${\frac{dF(z)}{dz}|}_{z=0}<0\;\text{and}\;{\frac{dF(z)}{dz}|}_{z=1}>0.$$

Therefore, *z* = 0 is the equilibrium point, and media and KOLs choosing the“not spread” strategy becomes the evolutionary stable strategy.

The dynamic changes in the strategies of media and KOLs under different conditions are illustrated in Fig 3c.

#### 3.2.3 Stability analysis of equilibrium points.

From Eq (13), the Jacobian matrix *J* of the system can be derived as follows:


$$J=[\begin{array}{ccc} \frac{\partial (dx/dt)}{\partial x} & \frac{\partial (dx/dt)}{\partial y} & \frac{\partial (dx/dt)}{\partial z}\\ \frac{\partial (dy/dt)}{\partial x} & \frac{\partial (dy/dt)}{\partial y} & \frac{\partial (dy/dt)}{\partial z}\\ \frac{\partial (dz/dt)}{\partial x} & \frac{\partial (dz/dt)}{\partial y} & \frac{\partial (dz/dt)}{\partial z} \end{array}]$$

$$=[\begin{array}{ccc} \left(1-2x)\cdot \{zA_{1}+yA_{2}+A_{3}\right\} & x(1-x)B_{1} & x(1-x)B_{2}\\ y(1-y)C_{1} & (1-2y)C_{2} & y(1-y)P_{22}\\ z(1-z)(P_{32}-L_{32}) & z(1-z)L_{31} & (1-2z)C_{3} \end{array}],$$

where


$$A_{1}=a(C_{11}+L_{13})(1-y)+(1-a)P_{12}+L_{12},\;A_{2}=(1-a)P_{11}+L_{11},\;A_{3}=(ay-1)(C_{11}+L_{13})+C_{12},\;B_{1}=(1-a)P_{11}+a(1-z)(C_{11}+L_{13})+L_{11},\;B_{2}=a(C_{11}+L_{13})(1-y)+(1-a)P_{12}+L_{12},\;C_{1}=P_{23}+L_{21}-L_{22},\;C_{2}=zP_{22}+x(P_{23}+L_{21}-L_{22})+P_{21}-C_{21}-L_{21},\;C_{3}=P_{31}-C_{31}+x(P_{32}-L_{32})+yL_{31}.$$

By solving Eq (13), the equilibrium points are determined as follows:


$$P_{1}=(0,0,0),\;P_{2}=(1,0,0),\;P_{3}=(0,1,0),\;P_{4}=(0,0,1),\;P_{5}=(1,1,0),\;P_{6}=(1,0,1),\;P_{7}=(0,1,1),\;P_{8}=(1,1,1).$$

$$P_{9}=(\hat{x},\hat{y},\hat{z}),$$

where $\hat{x},\hat{y},\hat{z}$ are the solutions to the following equations (denoted as Eq 14):

$$zA_{1}-yA_{2}+A_{3}=0,\;zP_{22}+xC_{1}+C_{2}=0,\;C_{3}=0.$$ (14)

Each of the first eight equilibrium points is substituted into the Jacobian matrix *J*, and the corresponding three eigenvalues for each equilibrium point are calculated. The results are summarized in Table 3.

**Table 3:** Eigenvalues of the Jacobian matrix.

Equilibrium point	$\lambda_{1}$	$\lambda_{2}$	$\lambda_{3}$
*P*_1_ = (0,0,0)	$C_{12}-C_{11}-L_{13}$	$P_{21}-C_{21}-L_{21}$	$P_{31}-C_{31}$
*P*_2_ = (1,0,0)	$-C_{12}+C_{11}+L_{13}$	$P_{21}+P_{23}-C_{21}-L_{22}$	$P_{31}+P_{32}-L_{32}-C_{31}$
*P*_3_ = (0,1,0)	$(1-a)(P_{11}-C_{11}-L_{13})+L_{11}+C_{12}$	$-P_{21}+C_{21}+L_{21}$	$P_{31}+L_{31}-C_{31}$
*P*_4_ = (0,0,1)	$(1-a)(P_{12}-C_{11}-L_{13})+L_{12}+C_{12}$	$P_{21}+P_{22}-C_{21}-L_{21}$	$-P_{31}+C_{31}$
*P*_5_ = (1,1,0)	$(1-a)(C_{11}+L_{13}-P_{11})-C_{12}-L_{11}$	$-P_{21}-P_{23}+L_{22}+C_{21}$	$P_{31}+P_{32}+L_{31}-L_{32}-C_{31}$
*P*_6_ = (1,0,1)	$(1-a)(C_{11}+L_{13}-P_{12})-C_{12}-L_{12}$	$P_{21}+P_{22}+P_{23}-L_{22}-C_{21}$	$-P_{31}-P_{32}+C_{31}+L_{32}$

When addressing online public opinion issues, the primary scenario involves government guidance, media and KOL dissemination, and ordinary netizens transitioning from a state of participation to non-participation, leading to the calming of public opinion. Therefore, the focus is on the stability analysis of points *P*_6_ = (1,0,1) and *P*_8_ = (1,1,1).

The Jacobian matrix *J*_8_ corresponding to point *P*_8_ = (1,1,1) satisfies the eigenvalue conditions given by (Eq 15) as follows:

$$\left\{ \begin{array}{c} (1-a)(-P_{11}-P_{12}-C_{11}+L_{13})-L_{11}-L_{12}-C_{12}<0,\\ -P_{21}-P_{22}-P_{23}+L_{22}+C_{21}<0,\\ -P_{31}-P_{32}-L_{31}+C_{31}<0. \end{array}\right.$$ (15)

The Jacobian matrix *J*_8_ for point *P*_8_ = (1,1,1) satisfies (Eq 16), which is the first method of Lyapunov. Therefore, the evolutionary strategy reaches an Evolutionary Stable Strategy (ESS) state. At this point: - The strategy of ordinary netizens tends toward “participation,” - The strategies of media and KOLs tend toward “dissemination.”

The eigenvalue conditions are:

$$\left\{ \begin{array}{c} \lambda_{1}<0,\\ \lambda_{2}<0,\\ \lambda_{3}<0. \end{array}\right.$$ (16)

If point *P*_6_ = (1,0,1) reaches the ESS stable state, it must also satisfy Eq (16). In this case: - The government’s strategy tends toward “guidance,” - The strategy of ordinary netizens tends toward “non-participation,” - The strategy of media and KOLs tends toward “dissemination.”

### 3.3 Numerical analysis of government guidance on online public opinion at different stages

Numerical simulations were implemented in MATLAB R2016a using the ode45 solver to numerically integrate the improved replicator dynamic system over the interval t∈[0,10]. Initial conditions were set as *x* = 0.3, *y* = 0.5, and *z* = 0.4, representing moderate baseline propensities for the government, ordinary netizens, and media/KOLs to adopt their respective positive strategies.

The parameter values were assigned based on a combination of theoretical reasoning and empirically grounded behavioral assumptions, with reference to recent studies on online public opinion dynamics [47,56]. In particular, costs, benefits, and loss terms were calibrated to reflect each agent’s role-specific risks, incentives, and communication constraints in public opinion events. For instance, higher guidance costs were assigned to government actors to reflect the institutional burden of proactive intervention, while media/KOLs were assigned higher payoff values for dissemination to capture their platform-driven exposure incentives. A full summary of parameter settings is provided in Table 4. These values ensure behavioral plausibility while supporting comparative analysis across varying strategic and temporal configurations.

**Table 4:** Parameter assignments for different agents in online public opinion events.

Parameter	Value	Parameter	Value	Parameter	Value
*C*_11_	7	*P*_12_	2	*P*_23_	1.5
*C*_12_	1.5	*C*_21_	1	*C*_31_	1.5
*L*_11_	3	*L*_21_	1	*L*_31_	1.5
*L*_12_	5	*L*_22_	1	*L*_32_	1.5
*L*_13_	10	*P*_21_	1.5	*P*_31_	3
*P*_11_	2	*P*_22_	1.5	*P*_32_	2

**Note:** *C*_*ij*_, *L*_*ij*_, and *P*_*ij*_ (where i=1,2,3) represent parameters for the government, ordinary netizens, and media/KOLs, respectively.

Simulation outputs include time-series trajectories that capture the evolution of strategies over time. All figures were generated using MATLAB’s built-in functions. The full simulation code and parameter configuration files have been made publicly available via the GitHub repository to facilitate reproducibility and further research.

Considering the impact of delayed government response in online public opinion events, simulations were conducted in MATLAB R2016a to analyze the strategic evolution of the three parties under different levels of delay: timely, moderately timely, and significantly delayed intervention.

**Low Delay Parameter: Strategy Evolution of the Three Parties**

**Fig 4:**
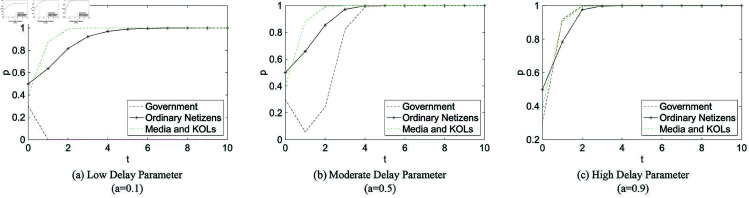
Simulation of three-party evolution with different delay parameters. *t*: time; *p*: probability of adopting a positive strategy.

A delay parameter of *a* = 0.1 corresponds to government intervention occurring during the early or latent stage of public opinion fermentation. The simulation results under this condition are presented in Fig 4a.

**Moderate Delay Parameter: Strategy Evolution of the Three Parties**

In the case of moderate delay (*a* = 0.5), government guidance takes place during the peak stage of public opinion development. The corresponding evolutionary dynamics are illustrated in Fig 4b.

**High Delay Parameter: Strategy Evolution of the Three Parties**

A higher delay value (*a* = 0.9) reflects government intervention at the fading stage of the opinion cycle. Fig 4c shows the resulting evolution of strategies for all three actors under this scenario.

The 3D evolutionary diagram illustrates the evolution process when the initial probabilities range from 0.1 to 0.9 with a step size of 0.2. In the visualization, blue, purple, and red lines represent the trajectories of ordinary netizens, media/KOLs, and the government, respectively.

To further examine the system’s dynamic behavior under diverse initial conditions, a series of simulations were conducted in MATLAB R2016a. The initial strategy adoption rates of government (*x*), netizens (*y*),and media/KOLs (*z*) were systematically varied over the interval [0.1,0.9] in steps of 0.2. For each initial state $\left(x_{0},y_{0},z_{0}\right)$, the system was numerically integrated over the time interval t∈[0,10].

Each trajectory captures the strategic evolution of the three actors over time, showing how the system converges or diverges from different starting conditions. The resulting trajectories were visualized in three-dimensional phase space, with color gradients encoding the initial state combinations. This approach reveals the stability basins and sensitivity of system dynamics to initial distributions.

**Fig 5:**
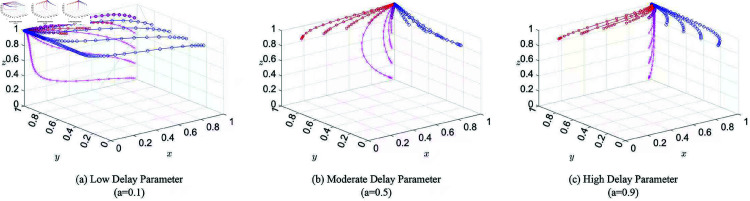
Three-dimensional evolutionary trajectories of strategy adoption under different delay parameters. Each subfigure illustrates the three-dimensional evolutionary trajectories of strategy adoption probabilities (*x*, *y*, *z*), corresponding to the government, ordinary netizens, and media/KOLs, respectively. Red lines with square markers represent trajectories starting from varying initial values of *x* (government), with *y* and *z* fixed. Blue lines with circle markers show varying initial *y* (netizens), and magenta lines with cross markers reflect varying initial *z* (media/KOLs). All trajectories converge toward a stable equilibrium, demonstrating how initial conditions for each agent influence the evolutionary path.

Fig 5 presents the overall results. The trajectories suggest that the system consistently evolves toward a stable equilibrium across a broad range of initial values, highlighting the robustness of the strategic dynamics under the given parameter configuration.

Specifically, when the delay parameter *a* = 0.1, the evolutionary stable strategy is (0,1,1), whereas for *a* = 0.5 and *a* = 0.9, the stable outcome shifts to (1,1,1).

Based on the 3D visualization and real-world considerations, we selected a representative initial state of *x* = 0.3, *y* = 0.5, and *z* = 0.8 to further examine convergence behavior. The degree of evolutionary convergence under different delay parameters is shown in Table 5. (MATLAB computes with 15-digit precision; values here are rounded to four decimal places. The final row in each column indicates the time at which convergence was achieved.)

**Table 5:** Convergence levels of three-party evolution under different delay parameters.

*t*	a=0.1			a=0.5			a=0.9		
0	0.3000	0.5000	0.8000	0.3000	0.5000	0.8000	0.3000	0.5000	0.8000
1	0.0142	0.7311	0.9794	0.1882	0.7633	0.9810	0.9990	0.8909	0.9872
2	0.0031	0.8807	0.9986	0.4954	0.9324	0.9990	0.9998	0.9900	0.9996
3	0.0015	0.9527	0.9999	0.8991	0.9910	1.0000	1.0001	0.9992	1.0000
4	0.0010	0.9821	1.0000	0.9898	0.9992		1.0001	0.9999	
5	0.0008	0.9933		0.9991	1.0000		1.0005	1.0000	
6	0.0006	0.9976		1.0000			1.0001		
7	0.0005	0.9991		1.0001			1.0006		
8	0.0000	0.9997		0.9999			1.0002		
9		0.9999		1.0006			1.0001		
10		1.0000		0.9995			0.9998		
11				1.0000			1.0000		

From Table 5, the government’s convergence times under different delay parameters are *t*_1_ = 8 for *a* = 0.1, *t*_2_ = 11 for *a* = 0.5, and *t*_3_ = 11 for *a* = 0.9. The government thus converges fastest under low delay conditions, with higher and moderate delays resulting in similar but slower trajectories.

Ordinary netizens converge at *t*_4_ = 10 (*a* = 0.1), *t*_5_ = 5 (*a* = 0.5), and *t*_6_ = 5 (*a* = 0.9). As illustrated in Fig 4, they converge significantly faster when the delay parameter is moderate or high, while the shortest delay yields the slowest convergence.

For media and KOLs, the convergence times are *t*_7_ = 4, *t*_8_ = 3, and *t*_9_ = 3 under *a* = 0.1, 0.5, and 0.9, respectively. Their strategies converge slightly faster under moderate and high delays than under low delay.

Furthermore, across all settings, the slope of the strategy curve for ordinary netizens is consistently steeper than that for media and KOLs, indicating a higher responsiveness to the evolving strategic environment.

## 4 Improved replicator dynamics model

### 4.1 Improved replicator dynamics equation

Based on the evolutionary game model described above, assume that the number of public opinion events where the government chooses the “guidance” strategy is *g*_1_, and the number of events where the government chooses the “no guidance” strategy is *g*_2_. Then:

$$x=\frac{g_{1}}{g_{1}+g_{2}}$$ (17)

As the timeline of public opinion events progresses, the rate of change ${\dot{g}}_{1}$ in the number of “guidance” decisions *g*_1_ is positively correlated with the number *g*_1_ and the expected payoff *U*_11_ of choosing the “guidance” strategy [57]:

$${\dot{g}}_{1}=\alpha_{1}\cdot g_{1}\cdot U_{11}$$ (18)

where:

- $\alpha_{1}$: the influence factor of the “guidance” strategy, indicating the effectiveness of the “guidance” strategy. In practical public opinion events, faster diffusion speed indicates stronger influence. A larger $\alpha_{1}$ reflects a stronger influence of the “guidance” strategy over the “no guidance” strategy.

Similarly:

$${\dot{g}}_{2}=\alpha_{2}\cdot g_{2}\cdot U_{12}$$ (19)

By differentiating Eq (17), the replicator dynamics equation for the government’s choice of the “guidance” strategy can be derived:

$$\dot{x}=\frac{g_{1}}{g_{1}+g_{2}}\left[\frac{{\dot{g}}_{1}}{g_{1}}-\frac{{\dot{g}}_{1}+{\dot{g}}_{2}}{g_{1}+g_{2}}\right]\;=x\left[\frac{\alpha_{1}\cdot g_{1}\cdot U_{11}}{g_{1}}-\frac{\alpha_{1}\cdot g_{1}\cdot U_{11}+\alpha_{2}\cdot g_{2}\cdot U_{12}}{g_{1}+g_{2}}\right]\;=\alpha_{1}x(1-x)\left(U_{11}-\frac{\alpha_{2}}{\alpha_{1}}U_{12}\right)$$ (20)

Similarly, the replicator dynamics equations for ordinary netizens and media/KOLs choosing the “participation” and “dissemination” strategies are:

$$\dot{y}=\beta_{1}y(1-y)\left(U_{21}-\frac{\beta_{2}}{\beta_{1}}U_{22}\right)$$ (21)

$$\dot{z}=\gamma_{1}z(1-z)\left(U_{31}-\frac{\gamma_{2}}{\gamma_{1}}U_{32}\right)$$ (22)

where:

- $\beta_{1}$: influence factor of the “participation” strategy for ordinary netizens, indicating the relative influence strength of “participation” versus “non-participation.” A larger $\beta_{1}$ indicates that “participation” has a stronger influence than “non-participation.”
- $\gamma_{1}$: similarly reflects the influence factor for media and KOLs.

To simplify and generalize the dynamic system, we define three incentive ratios: $\lambda_{1}=\frac{\alpha_{2}}{\alpha_{1}}$, $\lambda_{2}=\frac{\beta_{2}}{\beta_{1}}$, and $\lambda_{3}=\frac{\gamma_{2}}{\gamma_{1}}$. These parameters capture the relative strength between competing strategies within each agent group, and introduce behavioral asymmetries into the evolutionary process.

Specifically, $\lambda_{1}$ reflects the incentive relationship between the government’s “no guidance” and “guidance” strategies. When $\lambda_{1}>1$, the “no guidance” strategy exerts a suppressive effect on “guidance”—that is, the presence of inaction undermines the perceived necessity or effectiveness of proactive intervention. This may occur in practice when governments delay responses due to reputational risk, institutional inertia, or fear of public backlash. Conversely, $\lambda_{1}<1$ implies a reinforcing effect: widespread inaction heightens the urgency of guidance, such as in crisis scenarios where silence triggers external pressure or internal mobilization.

The same logic applies to $\lambda_{2}$, which models how “non-participation” influences ordinary netizens’ willingness to engage. A high $\lambda_{2}$ represents a self-reinforcing disengagement pattern, where individual apathy discourages collective participation—commonly seen in environments of political fatigue or low trust. In contrast, a low $\lambda_{2}$ corresponds to reactive mobilization, where passivity among peers creates moral or social incentives to speak up.

Similarly, $\lambda_{3}$ governs the relationship between “non-dissemination” and “dissemination” among media and KOLs. When $\lambda_{3}>1$, restrained discourse deters further amplification—possibly due to censorship, reputational concerns, or a low perceived news value. When $\lambda_{3}<1$, silence may paradoxically intensify the incentive to disseminate, reflecting rebound effects in communication dynamics, where initial restraint gives way to concentrated bursts of information release.

Incorporating these terms, the improved replicator dynamics system is expressed as:

$$\left\{ \begin{array}{c} \dot{x}=\alpha_{1}x(1-x)(U_{11}-\lambda_{1}U_{12})\\ \dot{y}=\beta_{1}y(1-y)(U_{21}-\lambda_{2}U_{22})\\ \dot{z}=\gamma_{1}z(1-z)(U_{31}-\lambda_{3}U_{32}) \end{array}\right.$$ (23)

When $\lambda_{1}=\lambda_{2}=\lambda_{3}=1$, this system simplifies to the replicator dynamics equation proposed by Taylor and Jonker [46], i.e. Eq (13).

The introduction of incentive coefficients allows this model to bridge intra-group behavioral adjustment and inter-group strategy evolution, offering a more comprehensive account of strategic decision-making in public opinion events.

Traditional game-theoretic models, such as Zhang and Ji [58], assume complete rationality, limiting their applicability to real-world, uncertainty-laden communication environments. Although evolutionary game models (e.g., Feng *et al*. [59]) account for bounded rationality, they often overlook intra-group behavioral influence, reducing the realism of their replicator dynamics.

By contrast, the present model not only preserves bounded rationality assumptions but also captures the internal strategic incentive structures within each group. This leads to higher theoretical granularity and better reflects the adaptive processes observed in empirical online discourse. A summary of the theoretical comparison is provided in Table 6.

**Table 6:** Model comparison results.

Method	Game type	Behavioral rationality	Accuracy of replicator dynamics rate	Application
J. Zhang and Ji [58]	Dynamic Game	Complete Rationality	—	Strategy Selection (Not Specific)
Feng *et al*. [59]	Evolutionary Game	Incomplete Rationality	Low	Strategy Selection (Not Specific)
This Study’s Method	Evolutionary Game	Incomplete Rationality	Relatively High	Strategy Selection (More Specific)

### 4.2 Numerical analysis of the evolutionary impact of different incentive coefficients

Since *U*_22_ = 0, the incentive coefficient $\lambda_{2}$ does not affect the evolution of the system. Based on the initial three-party evolution, government guidance in public opinion events is not necessarily better when initiated earlier. Considering actual scenarios, a delay parameter of *a* = 0.7 is chosen for numerical simulations to analyze the impact of different incentive coefficients on evolution. While the primary aim of this section is to explore the behavioral implications of varying incentive structures, the simulation results also serve as an implicit sensitivity analysis. By systematically varying $\lambda_{1}$ and $\lambda_{3}$, we are able to assess the robustness of the model’s evolutionary dynamics under different strategic conditions.

**Fig 6:**
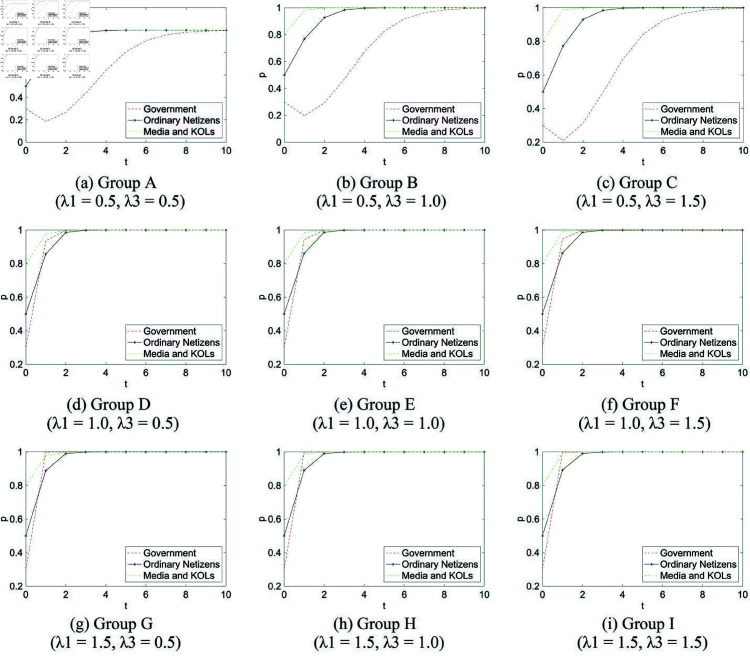
Three-party evolution simulation diagram. *t*: time; *p*: probability of adopting a positive strategy.

1. **Group A** ($\lambda_{1}=0.5$, $\lambda_{3}=0.5$)
  **Description:** Both the government’s “no guidance” and the media and KOLs’ “non-dissemination” behaviors strengthen the attractiveness of their respective action strategies, modeling a setting where passive stances stimulate active response. See Fig 6a.
2. **Group B** ($\lambda_{1}=0.5$, $\lambda_{3}=1.0$)
  **Description:** Government passivity encourages a shift toward guidance, while media and KOLs’ strategies remain uninfluenced by incentive asymmetries. See Fig 6b.
3. **Group C** ($\lambda_{1}=0.5$, $\lambda_{3}=1.5$)
  **Description:** Government inaction increases the relative value of guidance, whereas strong suppression from media and KOLs reduces the likelihood of dissemination. This represents a mixed-incentive environment. See Fig 6c.
4. **Group D** ($\lambda_{1}=1.0$, $\lambda_{3}=0.5$)
  **Description:** Government strategies are neutral, while lack of early dissemination encourages subsequent amplification by media and KOLs. See Fig 6d.
5. **Group E** ($\lambda_{1}=1.0$, $\lambda_{3}=1.0$)
  **Description:** All agent strategies evolve independently, with no incentive asymmetry between action and non-action choices. This setting corresponds to the baseline replicator dynamics without behavioral reinforcement or suppression. See Fig 6e.
6. **Group F** ($\lambda_{1}=1.0$, $\lambda_{3}=1.5$)
  **Description:** The government acts independently, but heightened suppression on the media and KOLs’ side reduces the strategic appeal of dissemination. See Fig 6f.
7. **Group G** ($\lambda_{1}=1.5$, $\lambda_{3}=0.5$)
  **Description:** The “no guidance” strategy weakens the incentive for intervention, while non-dissemination increases the appeal of information release among media and KOLs. See Fig 6g.
8. **Group H** ($\lambda_{1}=1.5$, $\lambda_{3}=1.0$)
  **Description:** Government guidance is inhibited by internal disincentives, while media and KOLs operate under neutral incentive conditions. See Fig 6h.
9. **Group I** ($\lambda_{1}=1.5$, $\lambda_{3}=1.5$)
  **Description:** Both the government and media and KOLs operate under suppressive incentive conditions, discouraging proactive guidance and dissemination alike. This models a high-inertia communication environment. See Fig 6i.

These groups provide insights into how different incentive coefficients influence the interaction and evolution of strategies among the government, ordinary netizens, and media/KOLs under delayed guidance conditions.

In the public opinion events with parameter assignments shown in Table 4, the degree of evolutionary convergence under different incentive coefficients is presented in Table 7 (MATLAB reads values with 15 significant digits, and here values are rounded to 4 decimal places; the last value in each column corresponds to the time of evolutionary convergence).

**Table 7:** Convergence levels of three-party evolution.

t	Group A ($\lambda_{1}=0.5$, $\lambda_{3}=0.5$ )			Group B ($\lambda_{1}=0.5$, $\lambda_{3}=1.0$ )			Group C ($\lambda_{1}=0.5$, $\lambda_{3}=1.5$ )		
0	0.3000	0.5000	0.8000	0.3000	0.5000	0.8000	0.3000	0.5000	0.8000
1	0.1848	0.7637	0.9700	0.1976	0.7685	0.9810	0.2095	0.7725	0.9880
2	0.2684	0.9228	0.9967	0.2931	0.9273	0.9990	0.3137	0.9307	1.0000
3	0.4390	0.9822	1.0000	0.4720	0.9838	1.0000	0.4975	0.9855	
4	0.6426	0.9971		0.6731	0.9973		0.6955	0.9979	
5	0.8075	0.9997		0.8278	0.9996		0.8421	0.9998	
6	0.9076	1.0000		0.9183	0.9999		0.9259	1.0000	
7	0.9583	0.9999		0.9634	1.0000		0.9669		
8	0.9819	1.0001		0.9840			0.9856		
9	0.9921	1.0002		0.9931			0.9938		
10	0.9966	0.9999		0.9970			0.9973		
11	0.9985	0.9998		0.9987			0.9989		
12	0.9994	1.0001		0.9995			0.9995		
13	0.9997	1.0005		0.9998			0.9998		
14	0.9999	0.9996		0.9999			0.9999		
15	1.0000	0.9997		1.0000			1.0000		
16		0.9999							
17		1.0000							
t	**Group D **($\lambda_{1}=1.0$, $\lambda_{3}=0.5$ )			**Group E **($\lambda_{1}=1.0$, $\lambda_{3}=1.0$ )			**Group F **($\lambda_{1}=1.0$, $\lambda_{3}=1.5$ )		
0	0.3000	0.5000	0.8000	0.3000	0.5000	0.8000	0.3000	0.5000	0.8000
1	0.9357	0.8575	0.9760	0.9423	0.8610	0.9855	0.9469	0.8639	0.9912
2	0.9997	0.9861	0.9984	0.9998	0.9866	0.9995	0.9998	0.9870	0.9999
3	1.0002	0.9989	0.9999	1.0000	0.9989	1.0000	1.0000	0.9990	1.0000
4	1.0000	0.9999	1.0000		0.9999			0.9999	
5	1.0003	1.0000			1.0000			1.0000	
6	0.9994								
7	0.9999								
8	1.0000								
t	**Group G **($\lambda_{1}=1.0$, $\lambda_{3}=1.5$ )			**Group H **($\lambda_{1}=1.5$, $\lambda_{3}=1.0$ )			**Group I **($\lambda_{1}=1.5$, $\lambda_{3}=1.5$ )		
0	0.3000	0.5000	0.8000	0.3000	0.5000	0.8000	0.3000	0.5000	0.8000
1	0.9990	0.8889	0.9784	0.9990	0.8909	0.9872	0.9991	0.8925	0.9924
2	0.9998	0.9897	0.9985	1.0000	0.9900	0.9996	1.0000	0.9902	0.9999
3	1.0000	0.9991	0.9999		0.9992	1.0000		0.9992	1.0000
4		0.9999		1.0000		0.9999			0.9999
5		1.0000				1.0000			1.0000

Based on Fig 6 and Table 5, the evolutionary speed and rate of the three parties under different combinations of incentive coefficients can be summarized as follows:

When the government’s incentive coefficient remains constant, increasing the suppressive influence of the “non-dissemination” strategy by media and KOLs—as seen in Groups ABC, DEF, and GHI—leads to accelerated convergence across all agents. This pattern is especially evident among ordinary netizens, who consistently demonstrate the fastest behavioral adjustment, followed by media and KOLs, and then the government. In real-world contexts, this may reflect how reduced media exposure or intentional silence from influential communicators drives individuals to quickly stabilize their opinions—either due to information fatigue, perceived irrelevance, or a lack of new signals. As the public receives fewer cues from media and digital elites, their engagement flattens, leading to earlier convergence of participation strategies.

A different pattern emerges when the incentive coefficient of media and KOLs is held constant and the government’s “no guidance” strategy becomes increasingly suppressive, as illustrated in Groups ADG, BEH, and CFI. In these settings, netizens again exhibit the highest responsiveness, likely reacting to perceived institutional absence or interpretive uncertainty. Governmental non-responsiveness in public opinion events often creates an informational vacuum, which can accelerate public speculation, social cue reliance, or rapid emotional convergence. Meanwhile, media and KOLs tend to adjust more slowly, possibly due to regulatory constraints, reputational considerations, or internal deliberation cycles that delay strategy shifts in high-uncertainty environments.

Interestingly, as the suppressive effect of “no guidance” surpasses a certain threshold, the convergence rate among ordinary netizens begins to plateau. Even though governmental suppression continues to increase, its marginal impact on public strategy adaptation weakens. This suggests a saturation effect in behavioral responsiveness—where early reactions eventually give way to disengagement, normalization, or strategic inertia. In contrast, the government and media and KOLs continue to adjust, albeit gradually, reflecting institutional delay in recalibrating to stabilized public discourse. This asymmetry underscores that while the public may shift rapidly and reach equilibrium early, institutional actors often lag behind, influenced by layered incentives and slower feedback loops.

Taken together, these findings highlight that the evolutionary pace of strategic adaptation is not uniform across actors. Ordinary netizens display higher sensitivity to incentive shifts, driven by decentralized decision-making and lower behavioral costs. In contrast, the strategies of media and government actors evolve more conservatively, constrained by organizational structure, reputational risk, and policy latency. These asymmetries offer important insights for designing more adaptive and timely intervention strategies in digital public opinion governance.

## 5 Conclusion and discussion

This study develops a three-party evolutionary game model to explore the dynamic interactions among government, media and KOLs, and ordinary netizens in online public opinion evolution. By introducing incentive coefficients into an improved replicator dynamics framework, the model captures the influence of intra-group behavioral reinforcement or suppression, enabling a more realistic representation of bounded rational decision-making in digital discourse environments.

Simulation results comparing different levels of government response delay suggest that early intervention does not always lead to greater stability. In contrast, a moderate delay—modeled as *a* = 0.7—tends to produce more balanced outcomes across actors. This finding aligns with practical experience in crisis communication [60], where interventions timed with the emotional trajectory of public attention often yield greater effectiveness than either premature reaction or prolonged silence.

Incentive configurations within each actor group also significantly affect convergence trajectories. The simulations show that when the government’s incentive coefficient is fixed (Groups ABC, DEF, GHI), increasing the suppressive influence of media and KOLs’ “non-dissemination” strategy accelerates convergence, particularly for ordinary netizens. In these settings, the order of responsiveness generally follows: netizens adapt fastest, followed by media and KOLs, and then the government. Conversely, when media and KOLs’ incentives remain constant (Groups ADG, BEH, CFI), and the government’s “no guidance” strategy becomes increasingly dominant, netizens still respond most rapidly, but the government may adjust faster than media actors. These findings suggest that public responsiveness tends to be higher in low-cost, decentralized decision environments [61], while institutional actors exhibit greater inertia due to regulatory, procedural, or reputational constraints [62].

Additionally, the simulations reveal a threshold effect: once the suppressive incentive of either “no guidance” or “non-dissemination” reaches a certain intensity, the behavioral response of netizens plateaus. Even if government or media actors continue to suppress action, the marginal effect on netizen adaptation diminishes, whereas institutional strategies still show incremental adjustment. This pattern may reflect saturation or desensitization, a phenomenon observed in long-duration online crises where early reactive engagement gives way to fatigue or disengagement [63].

These findings support the theoretical perspective that netizens act as a self-regulating force in the digital public sphere [64]. Their participation levels—represented by the variable *y*—respond dynamically to shifts in perceived credibility, transparency, and institutional behavior. Prior empirical studies in digital communication have emphasized similar dynamics, showing that online publics are more likely to engage when information appears timely, credible, and independent of manipulation [65]. The present model offers a formal representation of these mechanisms and shows how they may aggregate into systemic patterns of discourse stability or volatility.

Based on the simulation results, several concrete policy implications can be drawn. First, the model indicates that a moderate delay in governmental response (*a* = 0.5) yields more balanced and stable convergence outcomes than either immediate reaction (*a* = 0.1) or prolonged silence (*a* = 0.9). This suggests that interventions should be timed to align with the natural progression of public emotional engagement, rather than aiming for immediate disruption. Second, increasing the suppressive incentive of media/KOLs (e.g., $\lambda_{3}=1.5$) significantly accelerates convergence across all actor groups, particularly netizens. This points to the utility of coordinated signal suppression—such as limiting rumor amplification or encouraging silence during volatility peaks—under specific conditions. However, the diminishing returns observed beyond these thresholds warn against overreliance on suppression alone. Third, given the consistent finding that netizens respond faster than institutional actors, governments may benefit from adopting indirect, signal-oriented communication strategies early in the discourse cycle, allowing spontaneous public alignment before issuing formal guidance. Finally, policy frameworks should recognize that the effectiveness of interventions depends not just on timing, but on calibrated combinations of actor-specific incentives, cross-platform coordination, and reinforcement mechanisms that respect digital publics’ reflexivity.

Taken together, the results inform more nuanced governance strategies that go beyond one-sided narrative control. Rather than solely focusing on intervention timing or suppressive incentives, public opinion governance may benefit from designs that foster credibility, enable responsible amplification by media and KOLs, and respect the reflexive nature of public engagement. While governments retain a central role, their influence depends increasingly on alignment with intermediary actors and on trust-based communication rather than directive control.

Several limitations should be acknowledged. The model assumes behavioral homogeneity within each actor group and treats netizens and KOLs as independent agents, whereas in reality, public discourse is shaped by ideological divides, and KOLs often amplify content generated by netizens. These simplifications facilitate analytical tractability but overlook important feedback loops and group heterogeneity. Additionally, while the simulations are behaviorally grounded and informed by prior studies, they are not directly validated against real-world data. Future research could incorporate agent-based models or empirical event-level data to better capture opinion polarization, amplification dynamics, and real-time discourse evolution.
